# Comparative Genomics and Biosynthetic Cluster Analysis of Antifungal Secondary Metabolites of Three Strains of *Streptomyces albidoflavus* Isolated from Rhizospheric Soils

**DOI:** 10.3390/microorganisms12122637

**Published:** 2024-12-19

**Authors:** Adilene Gonzalez-Silva, Magali San Juan-Mendo, Gustavo Delgado-Prudencio, Juan Alfredo Hernández-García, Violeta Larios-Serrato, César Aguilar, Lourdes Villa-Tanaca, César Hernández-Rodríguez

**Affiliations:** 1 Departamento de Microbiología, Escuela Nacional de Ciencias Biológicas, Instituto Politécnico Nacional, Prol. Carpio y Plan de Ayala S/N, Mexico City CP 11430, Mexico; adigonzalezs@ipn.mx (A.G.-S.); magali031099@gmail.com (M.S.J.-M.); jahernandezga@ipn.mx (J.A.H.-G.); mvillat@ipn.mx (L.V.-T.); 2 Departamento de Medicina Molecular y Bioprocesos, Instituto de Biotecnología, Universidad Nacional Autónoma de México, Cuernavaca CP 62210, Mexico; gustavo.delgado@ibt.unam.mx; 3 Departamento de Bioquímica, Escuela Nacional de Ciencias Biológicas, Instituto Politécnico Nacional, Prol. Carpio y Plan de Ayala S/N, Mexico City CP 11430, Mexico; vlarios@ipn.mx; 4 Department of Chemistry, Purdue University, 575 Stadium Mall Dr. West Lafayette, Indiana, IN 47907, USA; caaguila@tec.mx

**Keywords:** *Streptomyces*, *Candida*, antifungal, antiSMASH, BGC, CORASON, candicidin, polyene, surugamide, synteny

## Abstract

*Streptomyces* is a genus of Gram-positive bacteria with high GC content. It remains attractive for studying and discovering new antibiotics, antifungals, and chemotherapeutics. *Streptomyces* genomes can contain more than 30 cryptic and expressed biosynthetic gene clusters (BGC) encoding secondary metabolites. In this study, three *Streptomyces* strains isolated from jungle rhizospheric soil exhibited supernatants that can inhibit sensitive and fluconazole-resistant *Candida* spp. The genomes of the strains *Streptomyces* sp. A1, J25, J29 ori2 were sequenced, assembled de novo, and analyzed. The genome assemblies revealed that the size of the genomes was 6.9 Mb, with linear topology and 73.5% GC. A phylogenomic approach identified the strains with high similitudes between 98.5 and 98.7% with *Streptomyces albidoflavus* SM254 and R-53649 strains, respectively. Pangenomic analysis of eight genomes of *S. albidoflavus* strains deposited in the Genomes database recognized 4707 core protein orthogroups and 745 abundant accessory and exclusive protein orthogroups, suggesting an open pangenome in this species. The antiSMASH software detected candicidin and surugamide BGC-encoding polyene and octapeptide antifungal secondary metabolites in other *S. albidoflavus*. CORASON software was used to compare the synteny, and the abundance of genes harbored in the clusters was used. In conclusion, although the three strains belong to the same species, each possesses a distinct genome, as evidenced by the different phenotypes, including antifungal and extracellular enzymatic activities.

## 1. Introduction

Soil and rhizosphere ecosystems are environments with a vast cultivable and non-cultivable microbial diversity, which participate in plant productivity, nutrient cycling, carbon storage, and biogeochemical cycles of carbon, nitrogen, sulfur, and phosphorus [1,2]. Plant growth-promoting rhizobacteria (PGPR) have gained importance as an alternative to sustainably promote crop growth and maintain soil fertility [3]. Several rhizospheric *Streptomyces* isolates establish mutualistic symbiosis with plants, promoting growth through the secretion of hydrolytic enzymes and excretion of phytohormones, siderophores, and secondary metabolites with antifungal activities [4].

The genus *Streptomyces* belongs to the phylum Actinomycetota, and it is a Gram-positive mycelial and spore-forming bacterium with high GC content, soil saprophytes with large and linear genomes (~8–10 Mb) [5], and often linear or circular plasmids [6]. The genomes of *Streptomyces* species harbor around 30 BGCs of secondary metabolites [7]. Under laboratory culture conditions, most of these clusters are strictly regulated, and many remain as “dormant” or “cryptic”. However, under specific environmental conditions, these clusters can be expressed [8,9]. Genome mining is a valuable tool for detecting BGCs encoding secondary metabolites of diverse chemical families, siderophores, and hydrolytic extracellular enzymes [10].

From an anthropocentric point of view, the secondary metabolites of *Streptomyces* have applications in health and agriculture as antibiotics, antifungals, chemotherapeutics, immunosuppressants, and anthelmintics, among others [7,11]. However, when it comes to the *Streptomyces* species, these substances also have a significant role in survival in highly competitive environments through the activation of biosynthesis of siderophores to iron absorption, UV-absorbing pigments to protect against ultraviolet radiation, and compounds with antioxidant activity [12]. Also, compatible solute, such as ectoin, prevents osmotic stress [13]. Meanwhile, γ-butyrolactone participates in chemical communication, conferring some adaptative advantages [14]. Other important secondary metabolites secreted with antifungal activity produced by *Streptomyces* spp. include broad chemical families of nucleoside analogs [15], nikkomycines [16] and polioxins [17], allylamines, and desotamides [18], which inhibit DNA replication, chitin synthetase, squalene epoxidase, and proteases, respectively. Also, compounds such as polyenes affect membrane activity by binding the ergosterol [19]. In the soil, *Streptomyces* secondary metabolites serve a chemical communication function and do not necessarily function as inhibitory agents against competing bacteria and fungi [20].

The organic nitrogen from microbial sources represents more than 80% of total soil nitrogen [21]. In particular, fungal and arthropod chitin, bacterial peptidoglycan, and microbial glycoproteins account for >60% of soil organic nitrogen [22,23]. *Streptomyces* spp. secrete many extracellular enzymes that recycle these macromolecules, producing simpler nitrogen-containing monomers such as *N*-acetylglucosamine (GlcNAc), *N*-acetylmuramic acid (MurNAc), and free amino acids [24]. Moreover, the amino sugars activate global sensory regulators of *Streptomyces* that induce the synthesis of secondary metabolites, such as GnrT and DasR protein activators. GlcNAc activates DasR and induces the production of antifungal metabolites; meanwhile, MurNAc induces antibacterial metabolites [14]. Other enzymes, such as cellulases, degrade cellulose, producing easily assimilable carbohydrates and stimulating soil microorganisms [25]. Also, multiple extracellular *Streptomyces* proteases are secreted. For example, *S. coelicolor* 56 annotated genome coding to 27 serine protease, 21 aminopeptidase, and eight metalloprotease genes [26]. In addition, *Streptomyces* can secrete other extracellular hydrolases, such as xylanase, chitinase, cellulase, amylase, and phosphatase [27].

In this study, the genomes of three *Streptomyces* strains with antifungal, enzymatic, and metal chelation abilities were sequenced, and BGC encoding to putative secondary metabolites with antifungal activity and extracellular enzymes genes was detected and related to phenotypic features.

## 2. Materials and Methods

### 2.1. Streptomyces Isolation, Culture, and Molecular Identification

*Streptomyces* strains were isolated and axenized from the jungle rhizospheric soil of Palenque, Chiapas, Mexico, conserved in the collection of the Laboratorio de Biología Molecular de Bacterias y Levaduras (LBMByL), Escuela Nacional de Ciencias Biológicas (ENCB), Instituto Politécnico Nacional (IPN). Also, the strains were deposited in the Colección de Microorganismos of Centro Nacional de Recursos Genéticos, Instituto Nacional de Investigaciones Forestales, Agrícolas y Pecuarias, Mexico. The *Streptomyces* sp. 102, 116, and J22 with no antifungal activity were included as negative controls in phenotypic tests. *Streptomyces* sp. A1, J25, and J29 ori2 with antifungal activity were recognized in this work as different strains of *Streptomyces albidoflavus*. From the rhizospheric soil samples, the bacteria were isolated by conducting the decimal serial dilution method using solid GAE medium: 20 g/L Glucose, 1 g/L asparagine, 0.5 g/L yeast extract, 0.5 g/L K_2_HPO_4_·7H_2_O, 0.5 g/L MgSO_4_·7H_2_O, 0.1 g/L FeSO_4_·7H_2_O, 15 g/L agar, pH 7 [28]. The Petri dishes were incubated at 28 °C for 10–20 days. Once the characteristic colonial morphology of actinobacteria was recognized, the strains were reisolated to obtain axenic cultures. Conventional Gram staining was used for the description of microscopic morphology. The mycelia and spores of pure strains harvested from cultures in solid media were conserved at −70 °C using 30% glycerol as the cryoprotective agent.

Primary identification of the strains was by recognition of the typical actinobacterial colony morphology, and molecular identification was performed by sequencing of the 16S rRNA gene. The primers used were 27F (5′-AGTTTGATCCTGGCTCAG-3′) and 1492R (5′-ACGGCTACCTACCTTGTACGACTT-3′) [29]. Amplification was detected by 1% (*w*/*v*) agarose gel electrophoresis using ultraviolet (UV) fluorescence with ethidium bromide, followed by purification of the reaction product using the Zymo Research DNA Clean & Concentrator^TM^-5 Kit (Irvine, CA, USA). After purification, the amplified samples were sequenced by the Sanger method in the laboratories of Macrogen^®^, Seoul, Republic of Korea (http://www.macrogen.com/).

### 2.2. Candida spp. Cultures

Several sensitive (S) and fluconazole-resistant (R) *Candida* species were used in this work, including *Candida albicans* ATCC 10231 (S), *Candida krusei* ATCC 14423 (R), *Candida glabrata* CBS 138 (S), and *Candida glabrata* 43 (R). Yeasts were cultured in YPD medium (10 g/L yeast extract, 20 g/L peptone of casein, 20 g/L glucose), and yeast inhibition tests were assayed in Sabouraud Dextrose Agar (SDA) medium supplemented with 40 g/L glucose.

### 2.3. Fungal Susceptibility of Candida spp. Strains

Fungal sensitivity and resistance to fluconazole were performed according to the Clinical & Laboratory Standards Institute (CLSI) Reference Method for Broth Dilution Antifungal Susceptibility Testing of Yeasts M27-A3 [30].

### 2.4. Bioassay for Candida spp. Growth Inhibitory Activity of Streptomyces Strains

Pure *Candida* strains preserved in 30% glycerol were used as pre-inocula, which were prepared according to the CLSI Reference Method for Broth Dilution Antifungal Susceptibility Testing of Yeasts [30]. Subsequently, 100 µL of each *Candida* strain’s suspension were adjusted to an optical density of 0.5 at 600 nm and spread on SDA petri dishes.

*Streptomyces* spp. strains were seeded in massive culture on GAE solid medium and incubated at 28 °C for 20 days. After the incubation time, agar bites were cut with Oxford towers for an antibiotics assay from each of the *Streptomyces* cultures. Each agar bite was taken and placed on plates of SDA medium massively inoculated previously with the *Candida* strains mentioned above. *Streptomyces* growth was placed on top, so that no direct contact between the *Streptomyces* and the yeasts was produced, and only diffusible metabolites allowed *Candida* growth. The plates with the bites were refrigerated for 2 h for diffusion into the SDA plates of the metabolites produced by *Streptomyces*. Subsequently, the plates were incubated at 37 °C for 24 h. and the inhibition halos were measured with a vernier. Bites of an uninoculated GAE medium subjected to the same incubation treatments were used as negative inhibition controls.

### 2.5. Obtention and Lyophilisation of Streptomyces Supernatants

Each 125 mL flask with sterile GAE medium was inoculated with spores of each *Streptomyces* strain and incubated at 28 °C with constant orbital shaking at 150 rpm for 15 days. After incubation, the cultures were centrifuged at 13,000 rpm for 25 min, and the supernatants were collected in 50 mL conical tubes. The supernatants were filtered through a 0.45 and 0.22 µm membrane and then lyophilized. The lyophilized supernatants were dissolved in sterile water at a final concentration of 200 mg/mL. The resuspended supernatants were centrifuged at 13,000 rpm for 10 min and filtered again through 0.22 µm pore size membranes. The supernatants were stored at −70 °C until use.

### 2.6. Confirmation of Antifungal Activity of Supernatants

The CLSI standardized microdilution method M27-A3 was used to evaluate the inhibition profile of the previously obtained lyophilized supernatants. The antifungal activity against *Candida* strains was evaluated in the range of 19 to 10,000 µg/mL. The absorbance at 620 nm was obtained in a MultiSkan FC 3.1 (Thermo Scientific^TM^, Waltham, MA, USA), and the minimum inhibitory concentrations of each supernatant and strain were determined. Dose–response assays were performed in triplicate. Statistical analysis was performed by two-way ANOVA.

### 2.7. Statistical Analysis

For the agar inhibition bioassay, the Student’s *t*-test was applied with a confidence interval of 95%. The data shown represent the arithmetic average; error bars indicate standard deviations. The results of antifungal sensitivity and MICs determination of *Streptomyces* supernatants were analyzed through a two-way analysis of variance (ANOVA), followed by a Bonferroni post-test with a confidence interval >99%. Analyses were performed with GraphPad Prism version 10.0.0 for Windows, GraphPad Software, Boston, MA, USA, available on www.graphpad.com.

### 2.8. Library Preparation and Genome Sequencing of Streptomyces A1, J25, and J29 ori2

*Streptomyces* strains seeded on GAE solid medium were incubated at 28 °C for 4 days. After incubation, according to the manufacturer’s instructions, DNA was extracted with the ZR Soil Microbe DNA MiniPrep^TM^ kit from Zymo Research. A 1% agarose gel electrophoresis was performed to verify DNA integrity. The genomic DNAs in the gels were visualized using ethidium bromide. The genomic DNA of each strain was fragmented to 150 bp, and libraries were constructed and sequenced using the Illumina HiSeq short read platform by Novogene Corporation Inc., Sacramento, CA, USA.

### 2.9. De Novo Assembly and Genome Annotation

Before assembly, the raw data were quality-controlled using the FastQC v. 0.11.9 program [31]. Adequate-quality reads were used to assemble genomes de novo following the workflow of the SPAdes v. 3.13.0 software [32]. Quality control of the assembled genomes was performed with QUAST software v. 5.3 [33]. Annotation was performed using Rapid Annotation using Subsystems Technology v. 2.0 (RAST) [34]. The genomes were visualized using the Proksee online server (https://proksee.ca/) [35].

Phylogenomic analyses were performed with OrthoFinder software (v2.5.4), and the ortho groups were defined as a set of genes descended from a single gene in the last common ancestor of all the species under consideration [36]. Other related Streptomyces genomes available at NCBI (National Center for Biotechnology Information) were collected. The phylogenomic tree was constructed with IQ TREE 2 software [37]. The Ortho Average Nucleotide Identity was calculated with ANI Calculator by EZBioCloud (https://www.ezbiocloud.net/) [38].

### 2.10. Comparative Genomics

Reference genomes were also annotated using Rapid Annotation using the Subsystem Technology (RAST) server v. 2.0. Comparative genomics for the search of orthologues was performed with the OrthoVenn3 online server (https://orthovenn3.bioinfotoolkits.net/) [39].

### 2.11. Bioassay for the Production of Extracellular Hydrolytic Enzymes and Siderophore of Streptomyces

The qualitative assays for extracellular enzymes were carried out in solid media and incubated at 37 °C for five days [40]. The diameters of hydrolysis halos were measured, and solubilization indices (SI) were calculated according to the formula:

SI = Colony diameter + Halo zone diameter/Colony diameter

In particular, qualitative assays for extracellular cellulase detection were carried out on Congo red plates (10 g/L carboxymethylcellulose; 1 g/L KH_2_PO_4_; 0.5 g/L MgSO_4_·7H_2_O; 0.5 g/L NaCl; 0.01 g/L FeSO_4_·7H_2_O; 0.01 g/L MnSO_4_·7H_2_O; 0.3 g/L NH_4_NO_3_; 15 g/L agar; 0.2 g/L Congo red). The diameter of transparent halos was measured. Extracellular amylases were detected on starch agar (8 g/L dehydrated nutrient broth; 25 g/L soluble starch; 15 g/L agar). After incubation time, the plates were developed with Lugol solution. Extracellular chitinases were inoculated on chitin-yeast extract-salt medium (5 g/L colloidal chitin; 0.5 g/L yeast extract; 2 g/L K_2_HPO_4_; 1 g/L MgSO_4_·7H_2_O; 0.1 g/L FeSO_4_·7H_2_O). After the incubation time, the presence of chitinases was evidenced by adding 0.1% of Congo red solution to reveal hydrolysis halos [41]. Extracellular proteases were detected on skim milk agar (8 g/L dehydrated nutrient broth; 25 g/L skim milk powder; 15 g/L agar). A translucent halo around the colonies evidenced the presence of proteases.

The putative genes encoding hydrolytic enzymes were mined in the RAST-annotated genomes [34]. The online server Gpos-mPLoc [42] was used to confirm the extracellular location of the extracellular hydrolytic enzymes of Gram-positive bacteria.

Siderophore production was revealed with the universal assay, Chromium Azurol S (CAS). GAE medium was used as a base. CAS was prepared by dissolving 60.5 mg of chromium azurol S in 50 mL of distilled H_2_O. Ten mL of Fe^3+^ solution (162 mg FeCl_3_ in 83.3 µL/HCl in 100 mL distilled H_2_O) was also added. On the other hand, 72.9 mg hexadecyltrimethyl ammonium bromide (CTAB) was dissolved in 40 mL distilled H_2_O. A color change around the colonies with an orange halo pointed to siderophore production [43].

### 2.12. Prediction and Genomic Context of BGCs of Antifungal Metabolites

Prediction of antifungal putative BGCs was performed with bacterial antiSMASH version v. 6.0 [44]. To determine the organization and genomic context of the putative BGCs, CORe Analysis of Synthenic Orthologues (CORASON) software (https://github.com/nselem/corason, accessed on 20 October 2024) was employed [10]. During the analysis, the reference genomes (previously used in the phylogenomic identification) were included. The reference clusters used during comparative genomics analysis were downloaded from the Minimum Information about a Biosynthetic Gene cluster repository 3.0 (MIBiG) [45].

## 3. Results

### 3.1. Colonial Morphology of Streptomyces Strains

**Figure 1:**
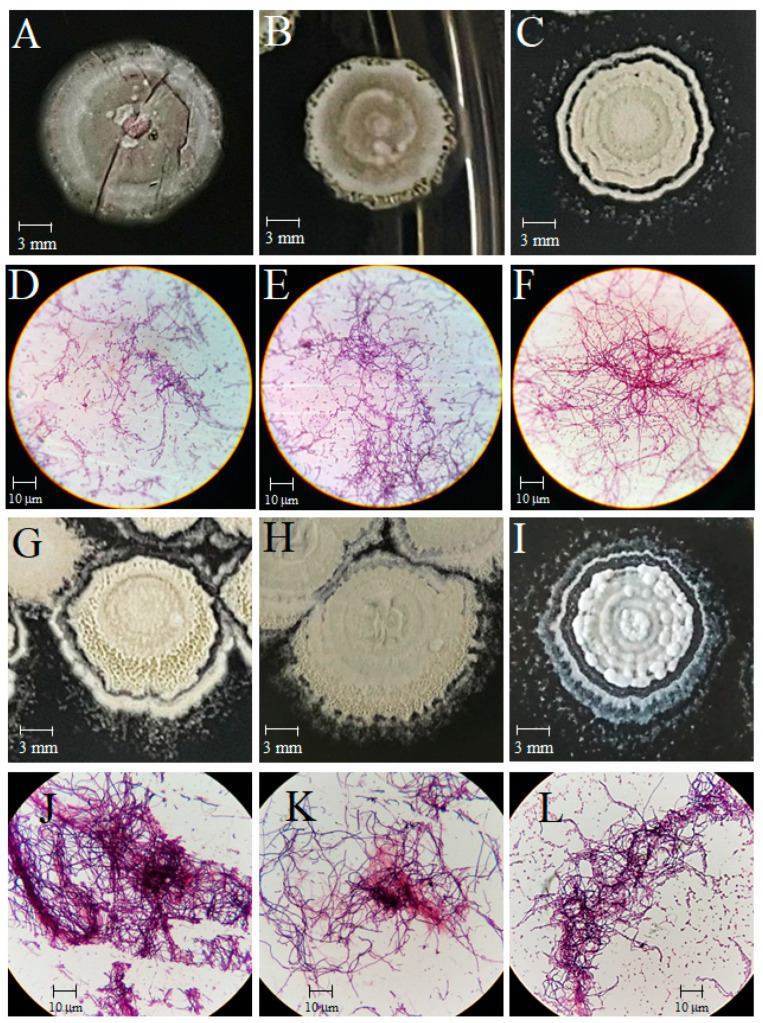
Colonial and microscopic morphologies of axenic cultures of *Streptomyces* sp. strains. (**A**–**C**) and (**D**–**F**), colonial and microscopic morphologies of *Streptomyces* sp. 102, 116-B, and J22, respectively; (**G**–**I**) and (**J**–**L**), colonial and microscopic morphologies of *Streptomyces* sp. A1, J25 and J29 ori2, respectively. Conventional Gram staining was used for description of microscopic morphology and observed at 1000×. Bars in panels (**A**–**C**) and (**G**–**I**) represent colony size in mm. Bars in panels (**D**–**F**,**J**–**L**) represent the mycelial diameter in μm.

The strains studied in this work were previously isolated from rhizosphere plants growing in jungle soils of south-eastern Mexico. The three strains that showed biological activity were incorporated after screening the LBMByL (ENCB-IPN) collection before this work. The purity of the strains was verified, and the characteristic colonial morphology was recognized, including circular colonies 4–8 mm in diameter with flat or convex elevation, filamentous edges, and a granular or powdery surface after 7 days of incubation. Particularly, isolate 116-B showed grey aerial and brown vegetative mycelia. Meanwhile, isolates 102, A1, J22, J25, and J29 ori2 showed white aerial and pale brown mycelia. Also, isolates J25 and J29 ori2 exhibited a brown diffusible pigment in the culture media (Figure 1). Also, typical microscopic Gram-positive mycelial morphology and spore chains were observed.

Phylogenetic identification based on the 16S rRNA gene revealed that isolates A1, J25 and J29 ori2 belong to the genus *Streptomyces* (Figure S1). However, it was not possible to assign them to species level with the similarity percentages obtained (96–98%) (Table S1).

### 3.2. Fungal Susceptibility of Candida spp. Strains to Determine Fluconazole Phenotype

The fluconazole sensitivity phenotype of *Candida* strains was determined according to the cut-off points established by CLSI, finding that *C. albicans* ATCC 10231 and *C. glabrata* CBS 138 have a sensitive phenotype, while *C. krusei* ATCC 14423 and *C. glabrata* 43 have a resistant phenotype (Figure S2).

### 3.3. Bioassay for Selection of Streptomyces spp. with Inhibitory Capacity for the Growth of Candida spp. Strains

**Figure 2:**
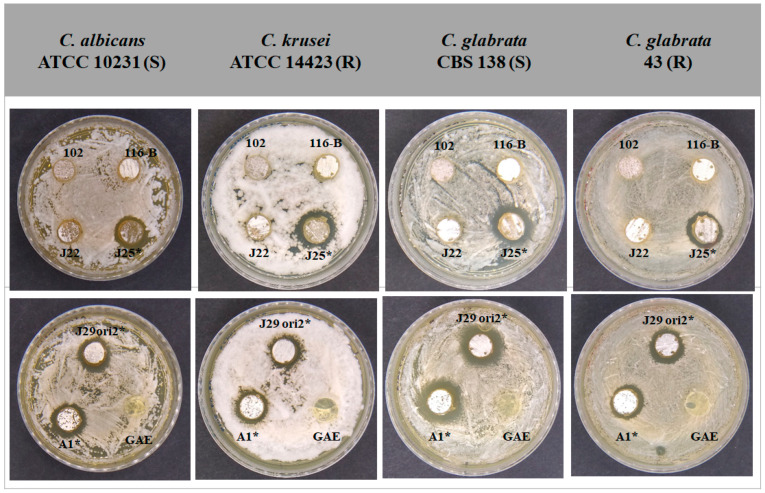
Bioassay of growth inhibition after 24 h at 37 °C of *Candida* spp. by metabolites produced by *Streptomyces* sp. strains. *Streptomyces* sp. 102, 116-B, and J22 did not exhibit antifungal activities on *Candida* spp. *Streptomyces* sp. A1 displayed 10–13 mm inhibition halos, *Streptomyces* sp. J25 displayed 10–12 mm inhibition halos and *Streptomyces* sp. J29 ori2 displayed 10–13 mm inhibition halos. GAE fresh medium was used as a negative control. Inhibition halos were measured after 24 h of incubation at 37 °C. The symbol * represents the statistically significant difference. Fluconazole sensitive (S) and resistant yeast (R).

SDA plates were inoculated and incubated with the yeasts, and the diameter of the inhibition halos of *Candida* spp. by *Streptomyces* strains was measured (Figure 2).

### 3.4. Determination of the Minimum Inhibitory Concentration of Supernatants

**Figure 3:**
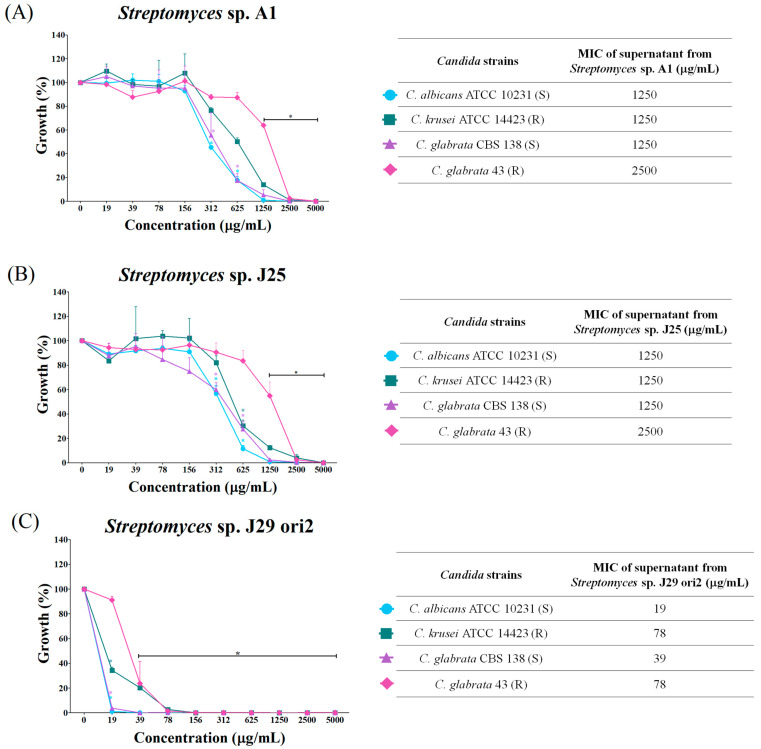
Dose–response effect of lyophilized supernatants of *Streptomyces* spp. and determination of minimum inhibitory concentrations on *Candida* spp. by the microdilution plate method. Panel (**A**), graph, and table of MICs obtained for the supernatant of *Streptomyces* sp. A1; (**B**), graph, and table of MICs obtained for the supernatant of *Streptomyces* sp. J25, and (**C**), graph, and table of MICs obtained for the supernatant of *Streptomyces* sp. J29 ori2. Figures and line in blue represent growth of *C. albicans* ATCC 10231; in green, *C. krusei* ATCC 14423; in purple, *C. glabrata* CBS 138; and in pink, *C. glabrata* 43. The symbol on the bar (*) represents statistically significant difference by two-way ANOVA analysis (*p* < 0.001). Fluconazole sensitive (S) and resistant yeast (R).

The lyophilized and rehydrated supernatants of *Streptomyces* spp. (200 mg/mL) were tested against strains of *Candida* spp. A dose–response effect between 19 and 5000 µg/mL was observed. The highest concentrations exhibited the smallest growth (Figure 3). The lyophilized supernatants of *Streptomyces* sp. A1 and J25 inhibited *Candida* species at MICs between 1250 and 2500 µg/mL (Figure 3A,B). Also, from the concentration of 1250 μg/mL onwards, a statistically significant difference was observed with respect to the growth control. The *Streptomyces* sp. J29 ori2 (Figure 3C) supernatant presented the most significant growth inhibitory capacity with MICs of 19, 39, 78, and 78 µg/mL for *C. albicans* ATCC 10231 (S), *C. glabrata* CBS 138 (S), *C. krusei* (R), and *C. glabrata* 43 (R), respectively. In this case, a statistically significant difference with respect to the growth control was observed at a concentration of 19 μg/mL.

### 3.5. Genome Sequencing, Assembly, and Annotation of S. albidoflavus Strains

All the reads analyzed by FastQC and filtering had a Phred scale >36. After assembly with SPAdes, and the coverage obtained was the 1×. Genome sizes were between 6.92 and 6.94 Mb, exhibiting typical linear topology, GC content (73.5%), numbers of coding genes between 6220 and 6257, and a total of contigs between 43 and 124 (Table S2).

The graphical representation of the strains’ genome (Figure S3) located the positions of non-encoding RNA, tRNA, rRNA, contigs, G-C content, and genes present in the genome. In addition, CRISPR regions and the origin of replication initiation (oriC) could be visualized. These figures also allowed comparison of the structural organization of the genes.

### 3.6. Genome-Based Taxonomic Identification of the Streptomyces Species

**Figure 4:**
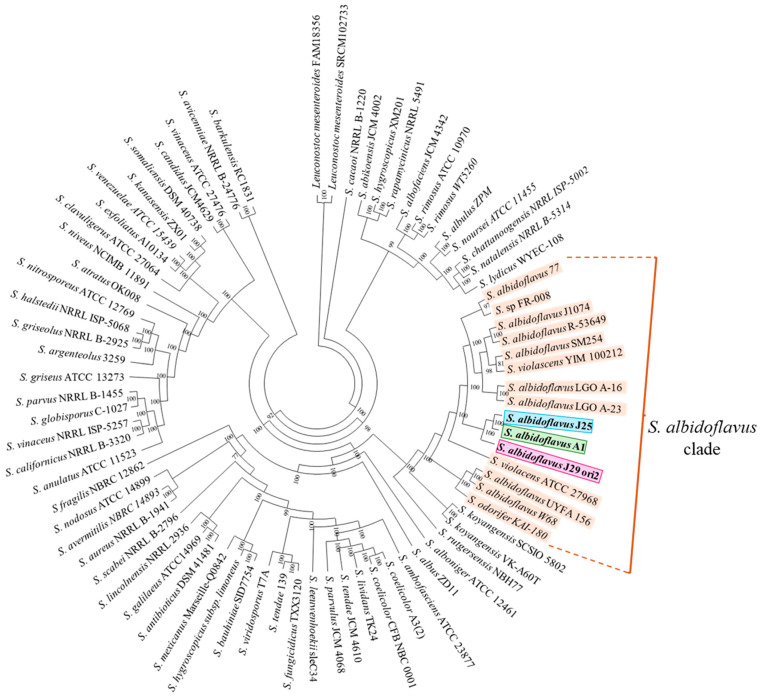
Phylogenomics identification tree by maximum likelihood of *Streptomyces albidoflavus* strains and related species. The core of 134 protein orthogroups concatenated were defined by Orthofinder. The amino acid substitution model was Q. insect + F + I + I + I + R10. The numbers in the nodes indicate the Ultra-Fast-bootstrap values with 1000 replicates. *Leuconostoc mesenteroides* strains were used as an outgroup.

The phylogenetic approach based on the analysis of the 16S rRNA only allowed the identification of *Streptomyces* strains to genus level (Figure S1). The phylogenomic analyses of the *Streptomyces* strains performed in Orthofinder associated all the strains with the *Streptomyces albidoflavus* species, which formed a consistent clade (Figure 4). An Ortho Average Nucleotide Identity (ANI) of entire genomes revealed high similitudes in the range of 98.58 and 98.71 among *S. albidoflavus* strains, but minor similitudes with other related *Streptomyces* species (Table 1). Both criteria support the assignation of the strains to the *S. albidoflavus* species. OrthoANI compares orthologous genes in the genomes of *S. albidoflavus* strains, allowing a more robust analysis of nucleotide identity.

### 3.7. Comparative Genomics Among S. albidoflavus Strains

**Figure 5:**
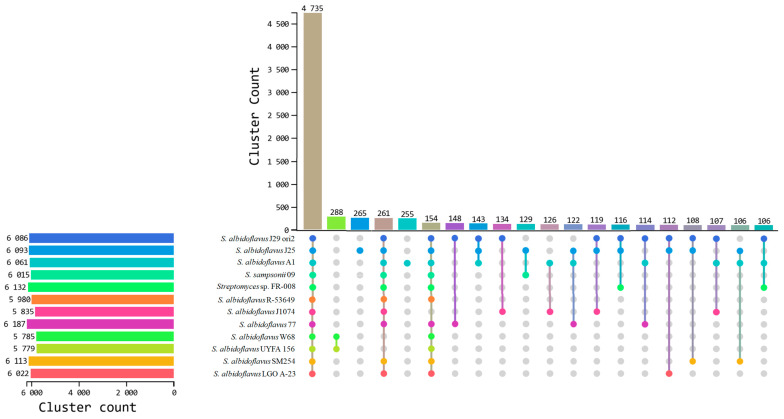
Upset plot of the comparative genomics to determine the number of orthogroups shared between *S. albidoflavus* strains. The colored bars on the left side represent the genomes of each *S. albidoflavus* strain and parents. The graph represents the number of orthogroups shared between them. The first bar is the number of orthogroups shared among all genomes or core genome of *S. albidoflavus*. The other columns show the orthogroups shared between groups of strains. For example, in the second column, *S. albidoflavus* W68 and *S. albidoflavus* UYFA 156 shared exclusively 288 orthogroups, and in the third column the blue dot represents the orthogroups unique to *S. albidoflavus* J25. The remaining columns can be interpreted in the same way. The gray dots mean the orthogroups are absent in the indicated genome. Comparative genomics and figure were performed on the OrthoVenn3 online server.

Orthovenn3 analysis recognized 4735 common orthogroups of three *S. albidoflavus* strains and nine strains from the database. Also, the strains contained between 106 and 288 accessory orthogroups. *S. albidoflavus* J25 and A1 strains harbored 265 and 255 exclusive orthogroups, respectively (Figure 5). In particular, only the strains *S. albidoflavus* J29 ori2, A1, and J25 included in this work shared 6014 common orthogroups and harbored 111, 7, and 24 exclusive orthogroups, respectively (Figure S4). Among these exclusive orthogroups a highlight was hypothetical proteins, glycosyltransferases involved in cell wall biogenesis and the transport of heavy metal-deduced proteins (Table S3).

### 3.8. Analysis of Extracellular Hydrolytic Enzymes and Siderophore Production of S. albidoflavus Strains

Phenotypic extracellular activities and their putative encoding genes for amylases, cellulases, chitinases, and proteases were identified in all three strains (Table 2). Frequently, more than one copy of each enzyme per genome was detected. More details of extracellular encoding enzymes, including enzyme class, function, contig ID, genome location, phenotypic activity, amino acid sequences, and solubilization indices (SI), were collected (Tables S4–S6, Figure S5).

In addition, the plate assay for siderophore production was positive, showing an orange halo around the colonies (Figure S6).

### 3.9. Analysis of BGCs of Metabolites with Antifungal Activity from S. albidoflavus Strains

The BGCs of secondary metabolite genes in *S. albidoflavus* J29 ori2, A1, and J25 genomes were detected by the antiSMASH bacterial version server. All three strains have BGC-encoding diverse chemical families of secondary metabolites with antibiotic, antifungal, antitumor, siderophore, and anticarcinogenic biological activities, among others (Tables S7–S9). According to phenotypical features, clusters encoding to antifungal compounds such as surugamides, polyenes such as candicidin, valinomycin, compound WS9326, and fluostatins were found in the three *S. albidoflavus* strains (Table 3).

### 3.10. Synteny Analysis of the BGC of Candicidin and Surugamide A/D of Streptomyces spp.

Based on the inhibitory activity of *S. albidoflavus* strains on *Candida*, synteny focused on analyzing candicidin and surugamide BGCs.

**Figure 6:**
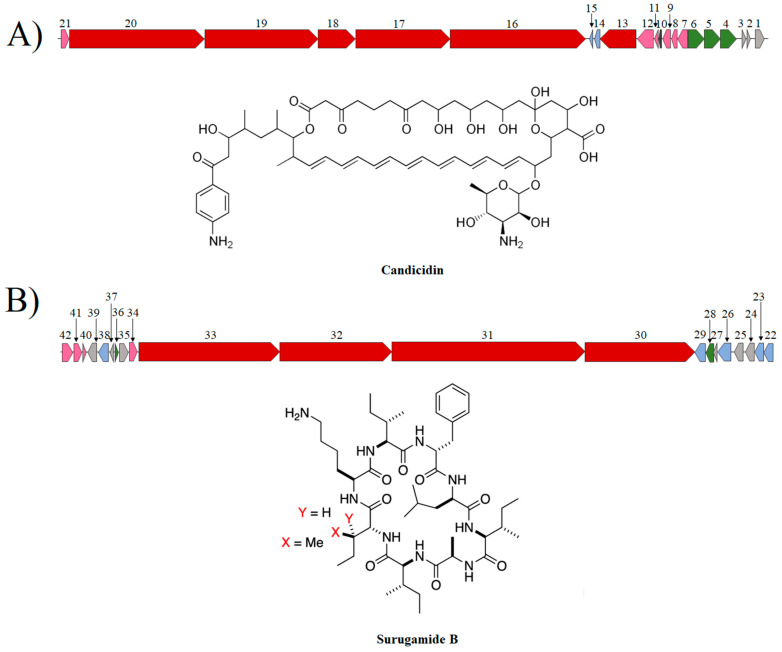
Organization of the candicidin (**A**) and surugamide (**B**) BGCs from *Streptomyces* sp. FR-008 and *S. albidoflavus* J1074, respectively. The arrows in red indicate the core genes of biosynthesis; in pink, the accessory genes; in green, the regulatory genes; in blue, the genes associated with transport; and in gray, the genes with other functions. **Candicidin genes:** 1. *fscO*, putative FAD-dependent monooxygenase (1377 bp); 2. *pabC*, putative 4-amino-4-deoxychorismate lyase (744 bp); 3. *fscRI*, putative transcriptional activator (669 bp); 4. *fscRII* LuxR family transcriptional regulator (2829 bp), 5. *fscRIII* LuxR family transcriptional regulator (3045 bp); 6. *fscRIV* LuxR family transcriptional regulator (3018 bp); 7. *fscMI* glycosyltransferase, MGT family (1377 bp); 8. *fscMII*, DegT/ DnrJ/ Ery C1/ StrS aminotransferase (1059 bp); 9. *fscP* cytochrome P450 (1182 bp)*;* 10. *fscFE* ferredoxin (194 bp); 11. *fscTE* thioesterase type II (858 bp); 12. *pabAB* isochorismate synthase (2172 bp); 13. *fscA,* beta-ketoacyl synthase (5232 bp); 14. *fscTI* ATP binding protein (transport) (1008); 15. *fscTII* ABC-2 type transporter (720 bp); 16. *fscC* acetyl-CoA acetyltransferase (31,878 bp); 17. *fscB* malonyl CoA-acyl carrier protein transacylase (16,626 bp); 18. *fscF* beta-ketoacyl synthase (6150 bp); 19. *fscE* beta-ketoacyl synthase (23,316 bp); 20. *fscD* beta-ketoacyl synthase (28,653 bp) and 21. *fscMIII* NAD-dependent epimerase/dehydratase (1209 bp). **Surugamide genes:** 22. XNR_3438, ABC transporter, permease protein (711 bp); 23. XNR_3439, ABC transporter, permease protein (762 bp); 24. XNR_3440, amino acid ABC transporter amino acid-binding protein (990 bp); 25. XNR_344 1, secreted protein (1221 bp); 26. XNR_3442, major facilitator superfamily permease (1575 bp); 27. XNR_3443, hypothetical protein (309 bp); 28. XNR_3444, TetR family transcriptional regulator (612 bp); 29. XNR_3445, drug resistance transporter EmrB/QacA subfamily protein (1347 bp); 30. *surD*, non-ribosomal peptide synthetase (12345 bp); 31. *surC*, non-ribosomal peptide synthetase (23,076 bp); 32. *surB*, ATP-dependent valine adenylase (12,798 bp); 33. *surA*, non-ribosomal peptide synthetase (17,202 bp); 34. *surE*, alpha/beta hydrolase MppK (1356 bp); 35. XNR_3451, putative membrane protein (1098 bp); 36. *surR*, transcriptional regulator, GntR family (417 bp); 37. XNR_3453, hypothetical protein (372 bp); 38. XNR_3454, ABC transporter ATP-binding protein (942 bp); 39. XNR_3455, ABC transport system membrane protein (798 bp); 40. XNR_3456, MbtH domain-containing protein (258 bp); 41. *lipE*, abhydrolase_6 biosynthetic-additional (810 bp), and 42. XNR_3458, succinate-semialdehyde dehydrogenase (1428 bp).

A synteny analysis of the candicidin and surugamide BGC with the highest percentage of similarity was performed to determine the genomic context, similitudes, and differences among these putative antifungal encoding clusters (Figure 6). The candicidin biosynthetic cluster (138.2 kb) contained six PKS type I genes (*fscA*-*fscF*) encoding to modular polyketide synthase enzymes which synthesize the macrolactone skeleton. Each deduced PKS enzyme contains ketosynthase repeat domains (KS), acyl transferase (AT), an acyl carrier protein (ACP), and, optionally, ketoreductase (KR), dehydratase (DH), and enoyl reductase (ER) domains. Also, the cluster harbors another 15 biosynthetic, transport, and regulatory genes that contribute to the final structure of the polyene. *pabAB* and *pabC* genes encode for 4-amino-4-deoxycorismate (ADC) synthase and ADC lyase, which participate in the synthesis of PABA, a precursor molecule that bonds to ACP to initiate candicidin biosynthesis. The *fscTE* gene encodes for a type II thioesterase, possibly responsible for removing aberrant intermediates and maintaining normal levels of candicidin synthesis. Also, the cluster includes *fscTI-II*, *fscRI-IV*, and *fscO* genes encoding to ABC type transporters, LuxR regulatory proteins, and a putative FAD-dependent monooxygenase, respectively.

**Figure 7:**
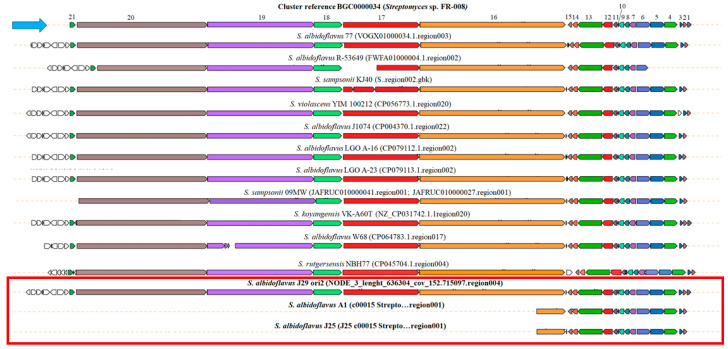
Synteny of BGC of candicidin of *S. albidoflavus* and related species. The figure was composed with the genetic contexts detected using *fscB* PKS and *pabAB* genes as a query in the CORASON software. The blue arrow points to the reference cluster, and the red box points to the *S. albidoflavus* A1, J25, and J29 ori2 genomes described in this work. **Candicidin genes:** 1. *fscO*, putative FAD-dependent monooxygenase (1377 bp); 2. *pabC*, putative 4-amino-4-deoxychorismate lyase (744 bp); 3. *fscRI*, putative transcriptional activator (669 bp); 4. *fscRII* LuxR family transcriptional regulator (2829 bp), 5. *fscRIII* LuxR family transcriptional regulator (3045 bp); 6. *fscRIV* LuxR family transcriptional regulator (3018 bp); 7. *fscMI* glycosyltransferase, MGT family (1377 bp); 8. *fscMII*, DegT/DnrJ/Ery C1/StrS aminotransferase (1059 bp); 9. *fscP* cytochrome P450 (1182 bp)*;* 10. *fscFE* ferredoxin (194 bp); 11. *fscTE* thioesterase type II (858 bp); 12. *pabAB* isochorismate synthase (2172 bp); 13. *fscA,* beta-ketoacyl synthase (5232 bp); 14. *fscTI* ATP binding protein (transport) (1008); 15. *fscTII* ABC-2 type transporter (720 bp); 16. *fscC* acetyl-CoA acetyltransferase (31,878 bp); 17. *fscB* malonyl CoA-acyl carrier protein transacylase (16,626 bp); 18. *fscF* beta-ketoacyl synthase (6150 bp); 19. *fscE* beta-ketoacyl synthase (23,316 bp); 20. *fscD* beta-ketoacyl synthase (28,653 bp) and 21. *fscMIII* NAD-dependent epimerase/dehydratase (1209 bp).

The CORASON analysis was performed with *fscB* (16,626 bp) and *pabAB* genes (2172 bp) encoding to type I PKS and isochorismate synthase, respectively, as query or center (Figure 7). The upload and download genes of the *fscB* and *pabAB* genes were ubicated according to the reference cluster of *Streptomyces* sp. FR-008. Clear differences between the clusters harbored in the three *S. albidoflavus* strains can be observed in the BGC synteny of candicidin. *S. albidoflavus* J29 ori2 has six PKSs genes (*fscA-fscF*) of candicidin cluster observed in the reference strain *S. albidoflavus* FR-008. Upstream of *fscD*, other sets of accessory genes of candicidin biosynthesis encoding amidases, oxidoreductases, and short-chain dehydrogenases complete the cluster. This candicidin cluster is broadly distributed among the *S. albidoflavus* clade and possibly encodes very similar candicidin structures.

On the other hand, *S. albidoflavus* A1 and *S. albidoflavus* J25 strains have only two PKS genes, a short *fscC*, and a conserved *fscA*. However, both strains maintain all regulatory genes, *fscRI-IV,* of the cluster. To our knowledge, the metabolite structure encoded by this cluster is unknown.

**Figure 8:**
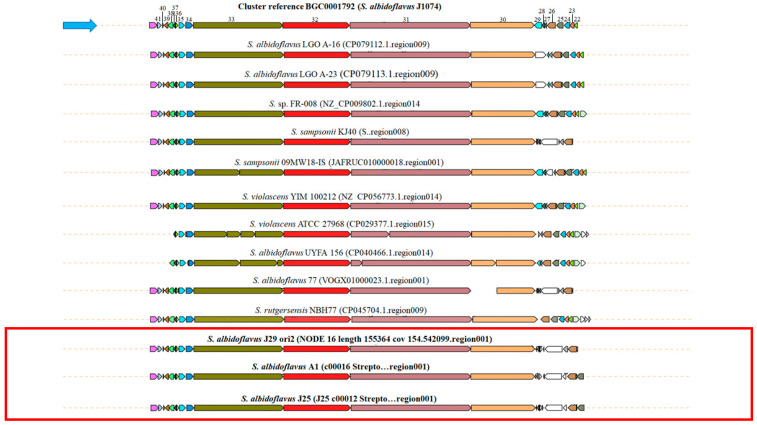
Synteny of biosynthetic gene clusters of surugamide A of *S. albidoflavus* and related species. The figure was composed with the genetic contexts detected using the *surB* gene as a query in the CORASON software. The data were taken from the antiSMASH comparison of the BGC query with the cluster reported in the MIBiG. The blue arrow points to the reference cluster, and the red box points to the *S. albidoflavus* A1, J25, and J29 ori2 genomes described in this work. **Surugamide genes:** 22. XNR_3438, ABC transporter, permease protein (711 bp); 23. XNR_3439, ABC transporter, permease protein (762 bp); 24. XNR_3440, amino acid ABC transporter amino acid-binding protein (990 bp); 25. XNR_3441, secreted protein (1221 bp); 26. XNR_3442, major facilitator superfamily permease (1575 bp); 27. XNR_3443, hypothetical protein (309 bp); 28. XNR_3444, TetR family transcriptional regulator (612 bp); 29. XNR_3445, drug resistance transporter EmrB/QacA subfamily protein (1347 bp); 30. *surD*, non-ribosomal peptide synthetase (12,345 bp); 31. *surC*, non-ribosomal peptide synthetase (23,076 bp); 32. *surB*, ATP-dependent valine adenylase (12,798 bp); 33. *surA*, non-ribosomal peptide synthetase (17,202 bp); 34. *surE*, alpha/beta hydrolase MppK (1356 bp); 35. XNR_3451, putative membrane protein (1098 bp); 36. *surR*, transcriptional regulator, GntR family (417 bp); 37. XNR_3453, hypothetical protein (372 bp); 38. XNR_3454, ABC transporter ATP-binding protein (942 bp); 39. XNR_3455, ABC transport system membrane protein (798 bp); 40. XNR_3456, MbtH domain-containing protein (258 bp); 41. *lipE*, abhydrolase_6 biosynthetic-additional (810 bp), and 42. XNR_3458, succinate-semialdehyde dehydrogenase (1428 bp).

The clusters containing the biosynthetic enzymes for synthesizing surugamide were found in the three strains of this study and many other *S. albidoflavus* of the Genome database. The percentages of similarity estimated by antiSMASH of BSG of *S. albidoflavus* A1, J25, and J29 ori2 with the reference cluster were high (90–100%), and the syntenies were very similar (Figure 8). The cluster has a total size of 82,464 bp and harbors 21 genes, including essential *surA*-*surD* and *surE* genes, which encode to NRPS for the synthesis of this cyclic peptide. A transcriptional regulator AcrR, a putative helicase, and an uncharacterized MFS-like transporter were also identified in all genomes.

## 4. Discussion

Secondary metabolites are non-essential small molecules produced by microorganisms. However, they have relevant functions in chemical communication, competition for nutrients, survival, antagonism, and mutualism [46]. From the anthropocentric point of view, secondary metabolites have applications in various fields and in the treatment of human, animal, and plant diseases.

In this work, *Streptomyces* sp. A1, J25, and J29 ori2 showed in vitro inhibitory activity against fluconazole-sensitive and resistant *Candida* spp. when their genomes were sequenced, assembled and annotated. In addition to the *Streptomyces* strains used in this work, other *S. albidoflavus* strains with inhibitory capacity also present MICs in the order of µg/mL [47], the same as fluconazole, suggesting that these species harbor a high diversity of secondary metabolites with inhibitory capacity against filamentous and yeast-like fungi [48,49,50]. The 16S rRNA-based identification could not identify the strains at the species level. However, the identification by the phylogenomic approach and ANI percentages suggest that the strains under study are associated with the *Streptomyces albidoflavus* clade. Despite the apparent taxonomic closeness of the strains, they exhibited different colonial morphologies, diffusible pigment production, phenotypical antifungal capabilities, and genetic secondary metabolite profiles. Similar findings were observed among closely related *Streptomyces griseus* strains with differences in pigmentation and secondary metabolite profiles, which appear to be strain-specific [51].

Typically, *Streptomyces* linear genomes are among the largest in prokaryotes. For example, *Streptomyces prasinosporus* harbor a large genome of 14.99 Mb, *S. coelicolor* a moderate genome of 8.6 Mb, and *Streptomyces gobiensis* a small genome of 5.7 Mb (GCA_039535455.1; [26,52]. In particular, the *S. albidoflavus* clade, including the three strains studied in this work, harbored relatively small genomes of around 6.8–7.3 Mb and maintained high paired similitudes above 98.5% (GCA_007896905.1, GCA_019286295.1 GCA_900171555.1). However, the comparison of structure genomes exhibited many chromosomal rearrangements. Beyond that, the pangenomic approach reveals an extensive core genome but also many exclusive genes harbored in the *S. albidoflavus* strains, which suggest frequent horizontal gene transference (HGT) events drawing an intraspecific genetic polymorphism and open pangenome similar to other *Streptomyces* [53]. Although extensive homologous recombination has been documented in *S. albidoflavus* strains [54], to our knowledge, no specific events of HGT have been studied in *S. albidoflavus*. These events in *Streptomyces* are relatively frequent in an evolutive time scale and may shape the vast diversity of secondary metabolite and antimicrobial peptide clusters, strain fitness, and adaptation to environmental changes of this genus [55,56,57,58,59].

*S. albidoflavus* strains have been isolated from different natural and artificial environments. *S. albidoflavus* UYFA 156 is a stem endophyte of *Festuca arundinacea* grass, and *S. albidoflavus* SM254 was isolated from mining sediments with high copper concentrations [60,61]. *S. albidoflavus* A1. J25 and J29 ori2 were isolated from rhizosphere and soil as were other similar strains [3,62,63,64].

Cellulose, starch, peptidoglycan, chitin, and proteins are abundant macromolecules of soil containing carbon and nitrogen [23]. Extracellular enzymes must hydrolyze all these relatively stable compounds to mobilize, recycle, and make them available as nitrogen and carbon sources for plants and other organisms [23,65]. By their origin, the three strains of *S. albidoflavus* expressed phenotypically extracellular enzymatic activities and harbored genes encoding extracellular amylases, cellulases, chitinases, and proteases. These enzymes are broadly distributed among *Streptomyces*, particularly in *S. albidoflavus* [66,67,68].

Like *S. coelicolor* and *Streptomyces lividans* genomes, *S. albidoflavus* strains harbor multigene chitinase families and synergic chitin-binding proteins promoting chitin degradation. *Streptomyces* chitininases have been used as biological control of fungal phytopathogens [24,69,70]. The peptidoglycan, chitin, and protein turnover introduce carbon and nitrogen sources to soil [71,72].

The three genomes of *S. albidoflavus* strains harbored putative genes and extracellular enzymes to degrade polysaccharides and proteins, producing substrates used as carbon and nitrogen sources. Multiple copies of extracellular cellulases, amylases, chitinases, and proteases encoding genes per strain were detected. This genetic redundancy has also been observed in other *Streptomyces* species’ genomes [26]. Genetic redundancy in bacteria is commonly subjected to purifying selection [73]. However, some genes evolve to sub-functionalization, neo-functionalization, or differential expression depending on selective pressures under stressful or stable environmental conditions [73]. In *S. coelicolor*, some redundant gene families that encode the same enzymes and specialized metabolite gene clusters (actinorhodin, isorenieratane, coelichelin, and a cryptic NRPS) are differentially expressed at the transcriptional level when grown in tween, glucose, or other carbon sources [74].

*Streptomyces* genomes commonly harbor many BGCs of secondary metabolite [7,75]. In particular, the genomes of *S. albidoflavus* A1, J25, and J29 ori2 strains harbor 13, 12, and 13 putative complete BGC of secondary metabolites, among which are antibiotics, antifungal, siderophores, antitumoral, pigments, and signally compounds. Although antifungal activity and siderophores of the strains were detected in vitro, other expressed or cryptic biological activities have yet to be detected. Genes encoding siderophores desferrioxamine B were demonstrated in vitro, and their possible encoding genes were detected in genomes. These iron-chelating NRPs exhibit antifungal abilities by competence for iron-limiting fungal growth [76]. *S. albidoflavus* strains produce several polyenes, non-polyenic, and phenolic compounds with antifungal properties against both filamentous and yeast fungi, and their application as a fungal biocontrol agent has been suggested [46,47,48,49,62,77,78,79].

The genomes of the strains in this work harbor BGC encode to polyenes with recognized antifungal activities such as candicidin, surugamides, and fluostatin. Among *Streptomyces* species, BGCs encoding polyene antifungals, such as pimaricin, nystatin, amphotericin B, and candicidin, are broadly distributed [19]. The candicidin biosynthesis gene cluster is widely distributed among *Streptomyces*, although their importance in ecology is poorly understood [80].

Candicidin is an amphipathic polyene containing a macrolactone ring of 59 carbons with a D-mycosamine residue covalently attached to ergosterol, which forms pores in the membrane, causing membrane disruption and, eventually, cell death [81].

According to the antiSMASH software, genomes of *S. albidoflavus* A1, J25, and J29 ori2 harbor BGC of candicidin similar to the BGC of the *Streptomyces* FR008 strain [82]; however a more detailed analysis with CORASON software demonstrated that only *S. albidoflavus* A1 and J29 ori2 completed the biosynthetic cluster. Particularly, *S. albidoflavus* A1 has a BGC encoding a short type 1 PKS encoded by *fscC*, suggesting that the synthesized metabolite is a different polyene to candicidin.

The first candicidin BGC was initially described in *Streptomyces griseus* IMRU 3570, but the clusters are widely distributed among different *Streptomyces* species [80,83]. This antifungal compound shares the structure of a macrocyclic ring and a mycosamine residue with other polyenes, such as nystatin, amphotericin B, and pimaricin, but also has a 4-aminophenyl residue, which gives an aromatic character to the molecule [75]. This study compared the organization of BGC of candicidin *S. albidoflavus*-related species with *Streptomyces* sp. FR-008 was used as a reference cluster [76]. Only *S. albidoflavus* J29 ori2 harbored the complete PKS cluster, which suggests that it encodes a similar candicidin molecule to reference and other *S. albidoflavus*, *S. rutgersensis*, and *S. violascens* BGCs [84]. Also, the upstream genes encoding amidases, oxidoreductases, and short-chain dehydrogenases implicated in the bond saturations and high resonance of carbon polyene skeleton are present in all the strains [85,86,87].

In the genomes of *S. albidoflavus* A1 and *S. albidoflavus* J25 strains, only a short *fscC* gene and a *fscA* encoding to PKS and regulatory genes *fscRI*-*IV* participate in the biosynthesis of an unknown polyene. Evolutionary studies of PKSs show that when the rest of the biosynthetic enzymes are not contiguous, they probably were acquired by HGT. Furthermore, the length of PKSs can vary depending on the environmental stresses to which the bacteria are subjected. Thus, PKSs can selectively incorporate modules, changing their length, and the resulting products have different structures and bioactivities [88]. An evolutive approach will be necessary to define if the short BGC of this polyene is a product of a phenomenon of evolution towards complexity or reductiveness.

Surugamides are natural cyclic NRPs from 6 to 10 amino acids belonging to the desotamides family, which exhibit antibiotic and antifungal activities and anticancer properties of cathepsin B through the inhibition of proteases [18,89,90]. NRPs are biosynthesized by a modular enzymatic assembly line catalyzed by NRPs synthases and cyclized by a cyclase belonging to the β-lactamase superfamily [91,92]. *S. albidoflavus* strains of this work maintain BGCs of surugamides with high synteny similarity with the reference BGC of *S. albidoflavus* J1074 and other strains from marine sediments [84,93,94]. Downstream *surD* gene of surugamide BGC, and other genes encoding hypothetical proteins, transporters, and transcriptional regulators were identified. Surugamide BGC has been described as cryptic [18,93]. However, many “silent” BGCs of *Streptomyces* are not expressed because the signal molecules or environmental elicitors are not commonly provided in the synthetic culture media. Many in vitro environmental conditions or co-cultures are required to express BGCs of secondary metabolites of some *Streptomyces* species [95,96,97].

Considering the results of the MICs of the supernatants with the arrangements presented by the BGCs of secondary metabolites with antifungal activity, it could be suspected that the difference in inhibitory capacity between each of the strains is given by the arrangements in the organization of their clusters, since this will, in one way or another, have a direct consequence on the final structure of the metabolite.

The identification in the genome of the secondary metabolites, such as siderophores and antifungals, such as polyenes and surugamides, among others, suggests that *S. albidoflavus* strains A1, J25, and J29 ori2 harbor significant potential for use as PGPR bacteria.

## 5. Conclusions

In conclusion, in this work, the genome of *Streptomyces albidoflavus* A1, J25, and J29 ori2 strains isolated from jungle soils were fully identified to species level by a phylogenomic approach because the 16S rRNA gene sequence is not sufficient for an adequate taxonomic assignment. The comparative genomics of the strains allowed the recognition of a core and accessory genome in each strain. Also, phenotypic assays and genome mining revealed BGCs and genes encoding to secondary metabolites with potential antifungal activities, extracellular hydrolytic enzymes, and siderophores. The synteny of BGCs of the metabolites candicidin and surugamide suggest a common origin. However, several genomic rearrangements probably explain the difference in inhibitory activity between the strains. It will be possible to relate the intraspecific diversity detected in the genomes to the specific phenotypic characteristics of each strain through comparative genomics and analysis of the arrangement of BGCs in phylogenomically related strains.

## Figures and Tables

**Table 1:** Average Nucleotide Identity by Orthology (OrthoANI) of whole genomes of *Streptomyces albidoflavus* strains and related species.

Reference Genome (GeneBank Access Number)	*Streptomyces* sp. A1	*Streptomyces* sp. J25	*Streptomyces* sp. J29 ori2
*Streptomyces albidoflavus* 77 (GCA_007896905.1)	98.62	98.62	98.60
*Streptomyces albidoflavus* J1074 (GCA_000359525.1)	98.62	98.61	98.65
*Streptomyces albidoflavus* LGO A-23 (GCA_019286295.1)	98.67	98.64	98.63
*Streptomyces albidoflavus* SM254 (GCA_001577385.1)	98.71	98.67	98.65
*Streptomyces albidoflavus* R-53649 (GCA_900171555.1)	98.68	98.64	98.66
*Streptomyces* sp. FR-008 (GCA_001431765.1)	98.61	98.59	98.60
*Streptomyces koyangensis* VK-A60T (GCA_003428925)	95.79	95.78	95.75
*Streptomyces odorifer* KAI-180 (GCA_013363465.1)	95.98	95.95	96.05
*Streptomyces vinaceus* ATCC 27476 (GCA_008704935.1)	78.25	78.26	78.13
*Streptomyces violascens* ATCC 27968 (GCA_009429105.1)	96.00	95.99	95.98

**Table 2:** Extracellular enzymes phenotypically expressed and their encoding genes of *S. albidoflavus* strains.

*S. albidoflavus* Strain	Phenotypic Extracellular Enzyme	Number of Encoding Genes	Solubilization Index (SI)
A1	Amylase	1	4.93
	Cellulase	3	2.28
	Chitinase	11	2.16
	Protease	22	3.26
J25	Amylase	1	5.23
	Cellulase	3	2.13
	Chitinase	11	2.17
	Protease	25	3.16
J29 ori2	Amylase	3	6.17
	Cellulase	2	2.22
	Chitinase	10	2.10
	Protease	28	4.24

**Table 3:** Putative BGCs of metabolites with antifungal activity from *S. albidoflavus* A1, J25, and J29 ori2.

Type	Activity	Most Similar Known Cluster	Similitude Percentage (%)
***S. albidoflavus* A1**			
T1PKS, NRPS	AF, AB	SGR PTMs	100
T1PKS, NRPS-like	AF	Candicidin	90
T2PKS	AF, ATM, AB	Fredericamycin A	60
NRPS, RRE-containing	AF	Surugamide A/Surugamide D	90
Siderophore	Q, AF	Desferrioxamin B	100
Siderophore	AF, AB	Ficellomycin	5
LAP, Thiopeptide	AF	Fluostatins M-Q	4
***S. albidoflavus* J25**			
T1PKS, NRPS	AF, AB	SGR PTMs	100
T1PKS, NRPS-like, NRPS, lanthipeptide Class II	AF	Candicidin	95
T1PKS	AF	Mediomycin A	28
T2PKS	AF, ATM, AB	Fredericamycin A	60
NRPS, RRE-containing	AF	Surugamide A/Surugamide D	100
Siderophore	AF, AB	Ficellomycin	5
Siderophore	Q, AF	Desferrioxamin B	100
LAP, Thiopeptide	AF	Fluostatins M-Q	4
***S. albidoflavus* J29 ori2**			
T1PKS, NRPS-like, NRPS, lanthipeptide Class II	AF	Candicidin	100
T1PKS, NRPS	AF, AB	SGR PTMs/SGR PTM Compound b-d	100
T2PKS, NRPS	AF, ATM, AB	Fredericamycin A	96
NRPS	AF, AB	Cyclofaulknamycin	75
NRPS, LAP	AF	Surugamide A/Surugamide D	100
NI-Siderophore	Q, AF	Desferrioxamin B	100
LAP, thiopeptide, RRE-containing	AF	Fluostatins M-Q	4

AF, antifungal; AB, antibacterial; ATM, antitumoral; Q, chelator; T1PKS, polyketide synthase type I; T2PKS, polyketide synthase type II; T3PKS, polyketide synthase type III; NRPS, non-ribosomal peptide synthetase; NRPS-like, NRPS-like fragment; LAP, Linear azol(in)e-containing peptides; RRE-containing, RRE-element containing cluster; RiPP-like, other unspecified ribosomally synthesized and post-translationally modified peptide product (RiPP); PKS-like, other types of PKS.

## Data Availability

The *S. albidoflavus* A1, J25 and J29 ori2 genome sequences herein generated were deposited in the GenBank. The identification number of the bioproject is PRJNA1171758. The accession numbers of the sequences are *S. albidoflavus* A1, *S. albidoflavus* J25 (SAMN44309340), and *S. albidoflavus* J29 ori2 (SAMN44309348).

## Supplementary Materials

The following supporting information can be downloaded at: https://www.mdpi.com/article/10.3390/microorganisms12122637/s1, Table S1: Percentage similitude of the 16S rRNA gene of *Streptomyces* strains. Table S2: Features of linear *S. albidoflavus* genomes. Table S3: Exclusive orthogroups in each of the *S. albidoflavus* genomes. Table S4–S6: Features of extracellular enzymes phenotypically expressed of *S. albidoflavus* strains. Table S7–S9. Putative BGC of secondary metabolite from *S. albidoflavus* A1, J25, and J29 ori2. Figure S1: Phylogenetic tree based on the 16S rRNA gene of *Streptomyces* strains. Figure S2: Sensitivity profiles and determination phenotype to fluconazole of *Candida* spp. Figure S3: Graphical representation of *S. albidoflavus* strains linear chromosomes. Figure S4: Venn diagram of the number of orthogroups shared between *S. albidoflavus* A1, J25, and J29 ori2. Figure S5: Bioassay to produce extracellular hydrolytic enzymes of *Streptomyces* strains. Figure S6: Bioassay to siderophore production of *Streptomyces* strains.
