# Supplementary material for: Comparative Genomics and Biosynthetic Cluster Analysis of Antifungal Secondary Metabolites of Three Strains of Streptomyces albidoflavus Isolated from Rhizospheric Soils
Source: Microorganisms. 2024 Dec 19;12(12):2637. doi: 10.3390/microorganisms12122637 (PMC11678301; doi:10.3390/microorganisms12122637)
Supplement: Supplementary file 1 [file microorganisms-12-02637-s001.zip › Supplementary material.pdf]

Table S1. Percentage similitude of the 16S rRNA gene of Streptomyces A1, J25, and J29 ori2 strains and their closest phylogenetic relatives.

**Actinobacteria**	**Reference strain**	**Access number**	**Similitude (%)**
**A1**	*Streptomyces globisporus* ICN-874	KX775307.1	99.3
	Streptomyces hydrogenans IMB16-195	MG190787.1	99.3
	Streptomyces violascens WZS031	MH482912.1	99.3
**J25**	*Streptomyces somaliensis* SL-3	KF973254.1	99.2
	Streptomyces fungicidicus 93	KF742498.1	99.2
	Streptomyces koyangensis B003	MG188671.1	99.2
**J29 ori 2**	*Streptomyces albidoflavus* UKM 02	KX266258.1	96.7
	Streptomyces albidoflavus IHBA 9992	KR085950.1	96.7
	Streptomyces sampsonii MDCE7	KP970680.1	96.7

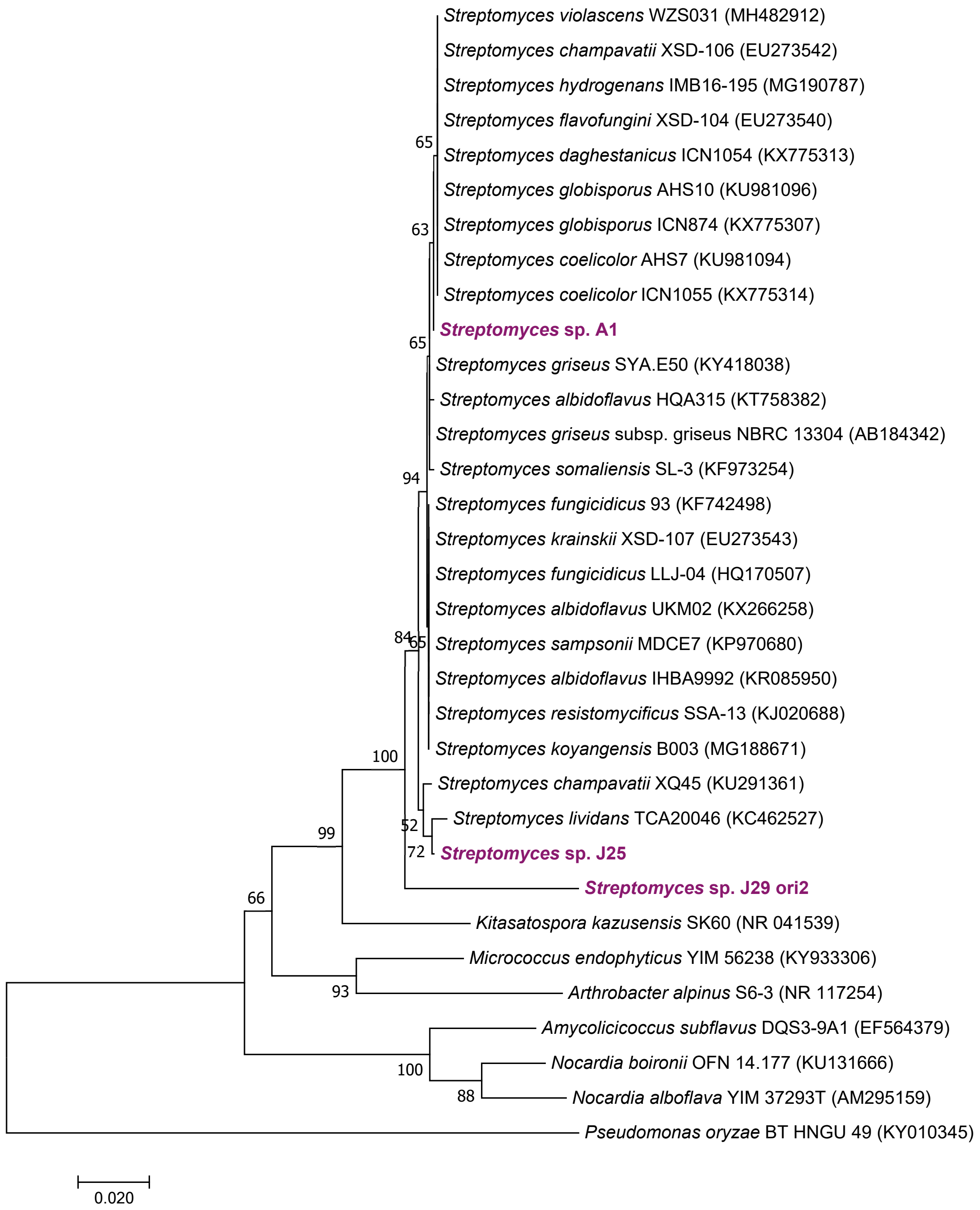

Figure S1. Phylogenetic tree based on the 16S rRNA gene of *Streptomyces* strains A1, J25, and J29 ori2 and their close phylogenetic relatives, reconstructed by the Neighbor-Joining method. The bar indicates the number of substitutions per site.

Bootstrap numbers greater than 50% are shown. *Pseudomonas oryzae* was used as outgroup.

A

Minimum inhibitory concentration (µg/mL)	*Candida* strains			
	C. albicans ATCC 10231	*C. krusei* ATCC 14423	*C. glabrata* CBS 138	*C. glabrata* 43
**Fluconazole**	8	64	8	>64
**CLSI phenotype**	S	R	S	R

B

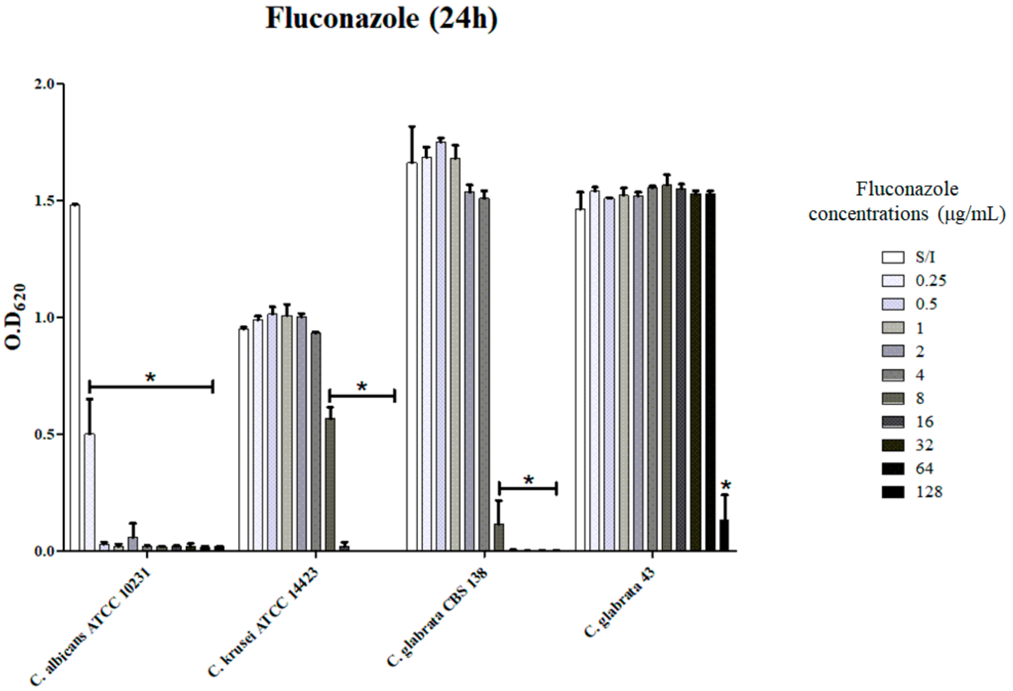

Figure S2. Sensitivity profiles and determination Phenotype to fluconazole of *C. albicans*, *C. glabrata* and *C. krusei*. Panel A, MICs and phenotype obtained for each of the *Candida* strains. Panel B, growth profile of *Candida* at increasing concentrations of fluconazole. The symbol on the bar (*) represents statistically significant difference by Two-way ANOVA (P< 0.001).

Table S2. Features of linear *S. albidoflavus* genomes.

**Feature**	***S. albidoflavus* A1**	***S. albidoflavus* J25**	***S. albidoflavus* J29 ori2**
Genoma size (Mb)	6.92	6.94	6.94
Contigs	55	43	124
N50	380779	454404	308571
Content of GC (%)	73.53	73.54	73.5
No. of coding genes	6220	6249	6257
oriC	1	1	1
rRNA	3	3	6
tRNA	68	68	69
tmRNA	1	1	1
ncRNA	37	24	39

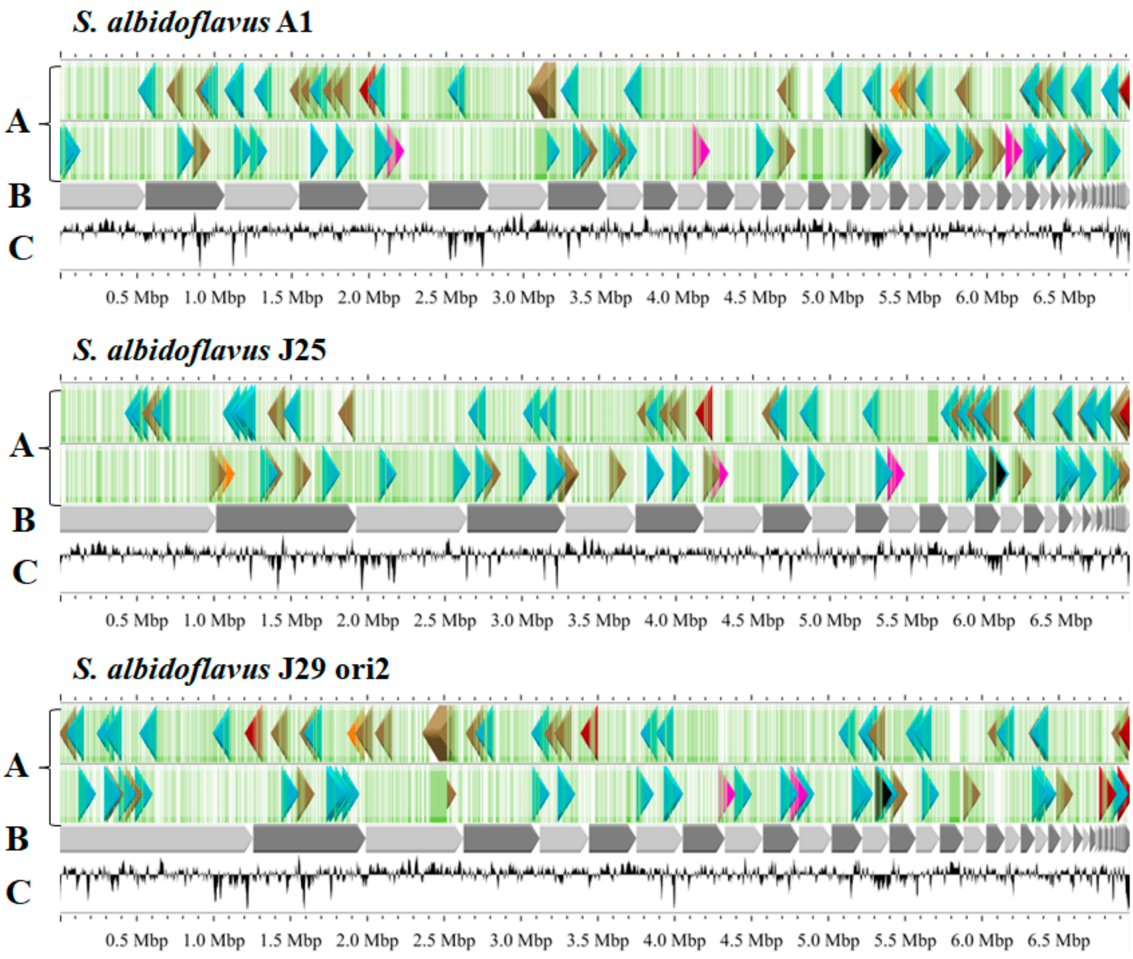

Figure S3. Graphical representation of *S. albidoflavus* strains linear chromosomes. A, CDS (green vertical lines), tRNAs (blue arrows), CRISPR regions (pink arrows), non-coding RNA regions (brown arrows), rRNAs (red arrows), *oriC* (black arrows); B, Contigs (gray arrows); C, GC content. Proksee online server was used to visualize chromosomes.

Table S3. Exclusive orthogroups in each of the *S. albidoflavus* genome

ID	Gene/Protein
***S. albidoflavus* A1**	
1883.2238.peg.406	Hypothetical protein
1883.2238.peg.3204	Hypothetical protein
1883.2238.peg.3549	Hypothetical protein
1883.2238.peg.3972	Hypothetical protein
1883.2238.peg.2815	Uncharacterized protein Sros-7085
1883.2238.peg.3710	Protein QmcA (possibly involved in integral membrane quality control)
1883.2238.peg.4747	Putative oxidoreductase
***S. albidoflavus* J25**	
1883.2239.peg.60	Putative oxidoreductase
1883.2239.peg.279	Two-component transcriptional response regulator, LuxR family
1883.2239.peg.2115	Integral membrane protein
1883.2239.peg.3026	Hypothetical protein
1883.2239.peg.3831	Translation elongation factor Tu
1883.2239.peg.4143	2,5-dioxovalerate dehydrogenase (EC 4.2.1.41)
1883.2239.peg.4176	Hypothetical protein
1883.2239.peg.4403	Hypothetical protein
1883.2239.peg.4567	Glycerophosphoryl diester phosphodiesterase (EC 3.1.4.46) periplasmic
1883.2239.peg.5554	Efflux ABC transporter, ATP-binding protein
1883.2239.peg.5977	Oxidoreductase, short-chain dehydrogenase/reductase family
1883.2239.peg.6023	N-acetylneuraminate synthase (EC 2.5.1.56)
***S. albidoflavus* J29 ori2**	
6666666.480568.peg.1866	Hypothetical protein
6666666.480568.peg.4411	Hypothetical protein
6666666.480568.peg.4960	Hypothetical protein
6666666.480568.peg.3944	Division initiation protein
6666666.480568.peg.5967	Hypothetical protein
6666666.480568.peg.264	Hypothetical protein
6666666.480568.peg.64	Hypothetical protein
6666666.480568.peg.4821	Hypothetical protein
6666666.480568.peg.2571	Hypothetical protein

6666666.480568.peg.2572	Hypothetical protein
6666666.480568.peg.2427	Hypothetical protein
6666666.480568.peg.4768	Hypothetical protein
6666666.480568.peg.4101	FIG214983: hypothetical protein (icw(2);Thiazole-_oxazole-modified_microcin_(TOMM)_synthesis)
6666666.480568.peg.5321	Pyruvate dehydrogenase E1 component like
6666666.480568.peg.3710	Modular polyketide synthase
6666666.480568.peg.6480	Hypothetical protein
6666666.480568.peg.2174	3-hydroxyacyl-CoA dehydrogenase (EC 1.1.1.35) / Enoyl CoA hydratase (EC 4.2.1.17)
6666666.480568.peg.4599	Hypothetical protein
6666666.480568.peg.4257	Hypothetical protein
6666666.480568.peg.4869	Mobile element protein
6666666.480568.peg.2784	Mobile element protein
6666666.480568.peg.6168	Mobile element protein
6666666.480568.peg.3080	Mobile element protein
6666666.480568.peg.1074	Mobile element protein
6666666.480568.peg.4549	Mobile element protein
6666666.480568.peg.5065	FIG01127234: large coiled-coil domains containing protein, actin-like
6666666.480568.peg.1624	Hypothetical protein
6666666.480568.peg.1535	Hypothetical protein
6666666.480568.peg.3345	Chromosome partition protein smc
6666666.480568.peg.6366	Transciptional regulator, CdaR-family
6666666.480568.peg.4191	Hypothetical protein
6666666.480568.peg.1797	Putative oxidoreductase
6666666.480568.peg.1431	Hypothetical protein
6666666.480568.peg.5995	Hypothetical protein
6666666.480568.peg.877	Hypothetical protein
6666666.480568.peg.4128	Hypothetical protein
6666666.480568.peg.4916	Hypothetical protein
6666666.480568.peg.4806	Cyclopropane-fatty-acyl-phospholipid synthase (EC 2.1.1.79)
6666666.480568.peg.2435	DNA translocase FtsK
6666666.480568.peg.3591	Putative membrane protein
6666666.480568.peg.4711	FIG01121710: hypothetical protein
6666666.480568.peg.4804	Hypothetical protein
6666666.480568.peg.1844	Hypothetical protein
6666666.480568.peg.4627	Quinone oxidoreductase (E.C 1.6.5.5)
6666666.480568.peg.1934	Putative transmembrane transport protein

6666666.480568.peg.4612	Tellurium resistance protein TerD
6666666.480568.peg.4602	Hypothetical protein
6666666.480568.peg.1964	Cys-tRNA(Pro) deacylase YbaK
6666666.480568.peg.3729	Hypothetical protein
6666666.480568.peg.4331	Two-component system sensor kinase
6666666.480568.peg.4607	Serine/threonine protein phosphatase
6666666.480568.peg.6457	Putative sensor-like histidine kinase
6666666.480568.peg.3874	FIG01122375: Hypothetical protein
6666666.480568.peg.4676	Hypothetical protein
6666666.480568.peg.6217	Alanyl-tRNA synthetase (EC 6.1.1.7)
6666666.480568.peg.4269	Transcriptional regulator, CdaR-family
6666666.480568.peg.1348	Siderophore synthetase small component, acetyltransferase
6666666.480568.peg.3268	Putative sensor and ATPase, component of G-protein-coupled receptor (GPCR) system
6666666.480568.peg.4749	Hypothetical protein
6666666.480568.peg.5441	FIG01120811 Hypothetical protein
6666666.480568.peg.605	Secreted protein
6666666.480568.peg.4669	Putative secreted protein
6666666.480568.peg.6436	Hypothetical protein
6666666.480568.peg.6069	Hypothetical protein
6666666.480568.peg.2267	2'-5' RNA ligase
6666666.480568.peg.4812	Putative cellulose-binding protein
6666666.480568.peg.4063	Hypothetical protein
6666666.480568.peg.2502	Hypothetical protein
6666666.480568.peg.6243	Putative phosphotransferase
6666666.480568.peg.2513	Hypothetical protein
6666666.480568.peg.5265	CAIB/BAIF family protein
6666666.480568.peg.3630	Thiamine pyrophosphate-requiring enzymes
6666666.480568.peg.3612	Putative membrane protein
6666666.480568.peg.3605	Ribonuclease D (E.C 3.1.26.3)
6666666.480568.peg.5194	Selenocysteine-specific translation elongation factor
6666666.480568.peg.4913	FIG01126127: hypothetical protein
6666666.480568.peg.4605	Hypothetical protein
6666666.480568.peg.624	Putative sensor and ATPase, component of G-protein-coupled receptor (GPCR) system
6666666.480568.peg.5068	Hypothetical protein
6666666.480568.peg.4486	Putative membrane protein

6666666.480568.peg.5113	Adenosylcobinamide kinase (EC 2.7.1.156) / Adenosylcobinamide-phosphate guanylyltransferase (EC 2.7.7.62)
6666666.480568.peg.3359	Putative regulatory protein
6666666.480568.peg.4429	Hypothetical protein
6666666.480568.peg.3806	Hypothetical protein
6666666.480568.peg.4167	Serine/threonine protein kinase
6666666.480568.peg.2202	Hypothetical protein
6666666.480568.peg.3495	Hypothetical protein
6666666.480568.peg.5554	Hypothetical protein
6666666.480568.peg.703	SSU ribosomal protein S2p (SAe)
6666666.480568.peg.4984	Unknown
6666666.480568.peg.1858	Dihydrolipoamide acyltransferase component of branched-chain alpha-keto acid dehydrogenase complex (EC 2.3.1.168)
6666666.480568.peg.3972	Flavohemoprotein (Hemoglobin-like protein) (Flavohemoglobin) (Nitric oxide dioxygenase) (EC 1.14.12.17)

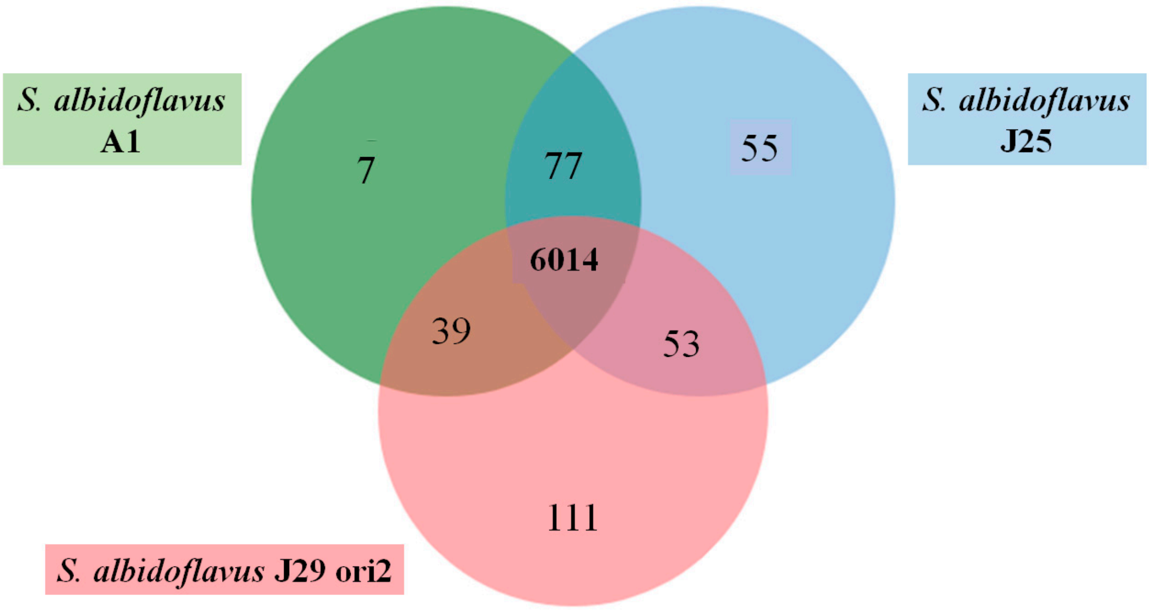

Figure S4. Venn diagram of the number of orthogroups shared between *S. albidoflavus* A1, J25, and J29 ori2. The core genome in the center included 6014

orthogroups, and in the periphery *S. albidoflavus* J29 ori2, A1, and J25 harbored 111, 7, and 24 exclusive orthogroups, respectively. Comparative genomics and Venn diagram were performed in jvenn.

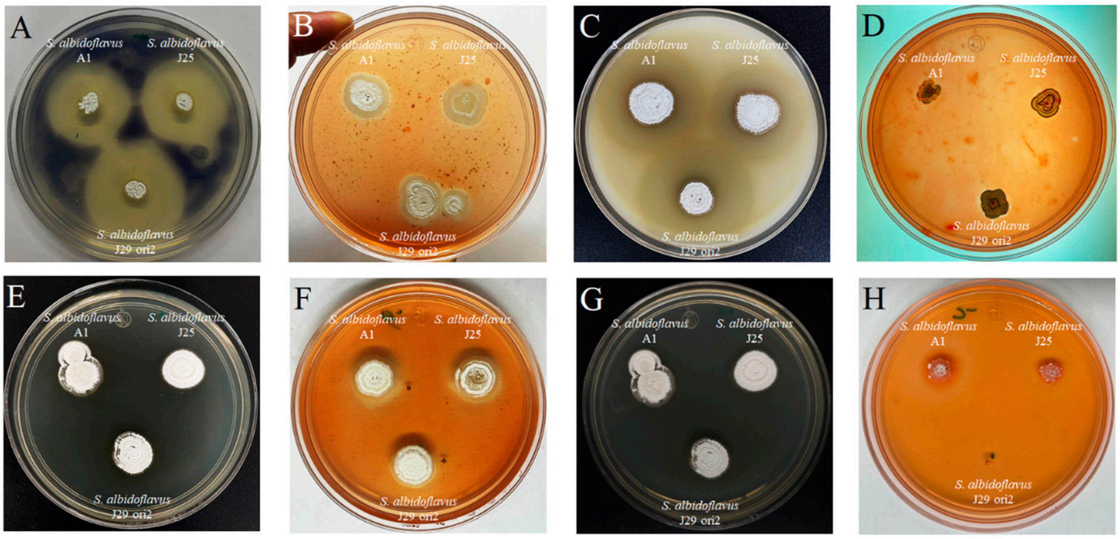

Figure S5. Bioassay to produce extracellular hydrolytic enzymes of *S. albidoflavus* strains. A, B, C, D, strains grown on starch, chitin, skim milk and CMC agar, respectively. E, F, G, H, strains grown on nutrient agar without starch, medium without chitin, nutrient agar without skim milk and medium without CMC, respectively, were used as growth controls. Hydrolysis halos and colony diameters were measured to determine solubility indices.

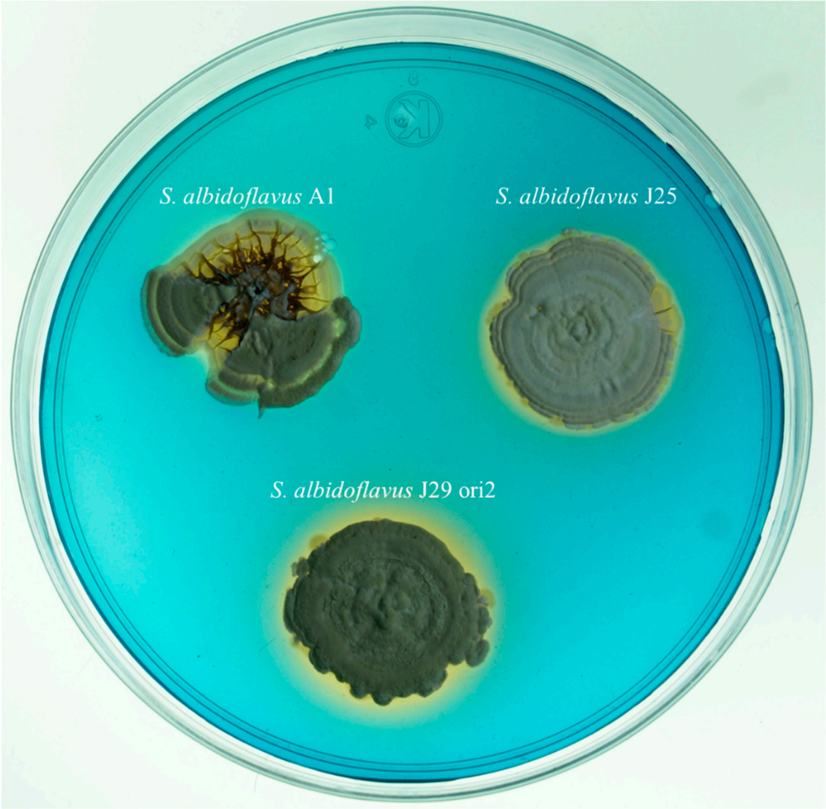

Figure S6. Bioassay to siderophore production of *S. albidoflavus* strains in GAE solid medium. A color change around the colonies with an orange halo pointed to siderophore production

Table S7. Putative BGCs of secondary metabolite from *S. albidoflavus* A1

**Type**	**Activity**	**Most similar known cluster**	**Similitude percentage (%)**
T1PKS, NRPS	AF, AB	SGR PTMs	100%
T1PKS	INMS	Sanglifehrin A	9%
T1PKS, NRPS, Lantipeptide- Class II, NRPS-like	PSC	Antimycin	100%
T1PKS	UK	-	N.D
T1PKS	UK	-	N.D
T1PKS	UK	-	N.D

T1PKS	UK	-	N.D
T2PKS	AF, ATM, AB	Fredericamycin A	60%
T3PKS	ATM	Herboxidiene	11%
NRPS, NRPS-like, terpene	IO, AB	Valinomycin / Montanastatin	13%
NRPS	Q, AB	Streptobactin	76%
NRPS	AB	Dechlorocuracomycin	16%
NRPS	AB	WS9326	7%
NRPS-like, T1PKS	AF	Candicidin	90%
NRPS, RRE-containing	AF	Surugamide A / Surugamide D	90%
NRPS	UK	-	ND
Siderophore	Q, AF	Desferrioxamin B	100%
Siderophore	AF, AB	Ficellomycin	5%
Lanthipeptide- Class III	AB	SAL-2242	100%
LAP, Thiopeptide	AF	Fluostatins M-Q	4%
Terpene	St	Hopene	76%
Terpene	Vo	Geosmin	100%
Terpene	AB	Julichrome Q3-3 / Julichrome Q3-5	25%
RiPP-like, terpene	Pi	Isorenieratene	75%
RiPP-like	UK	-	ND
RiPP-like	UK	-	ND
Butyrolactone, PKS-like, amglyccycl	S	7-deoxypactamycin	15%
Ectoine	Os	Ectoine	100%

*AF, antifungal; AB, antibacterial; INMS, immunosuppressant; PSC, piscicide; UK, unknown; ATM, antitumoral; IO, ionophore; Q, chelator; St, membrane component; Vo, volatile; Pi, pigment; S, signaling; Os, osmolyte; AC, anticarcinogenic; Mp, morphogenic; AM, metabolic activator; T1PKS, polyketide synthase type I; T2PKS, polyketide synthase type II; T3PKS, polyketide synthase type III; NRPS, non-ribosomal peptide synthetase; NRPS-like, NRPS-like fragment; LAP, Linear azol(in)e-containing peptides; NI, NRPS-independent; RRE-containing, RRE-element containing cluster; RiPP-like, other unspecified ribosomally synthesized and post-translationally modified peptide product (RiPP); PKS-like, other types of PKS.

Table S8. Putative BGCs of secondary metabolite from *S. albidoflavus* J25

**Type**	**Activity**	**Most similar known cluster**	**Similitude percentage (%)**
T1PKS, NRPS	AF, AB	SGR PTMs	100%
T1PKS	AB	Azalomycin F3a	34%
T1PKS, NRPS-like, NRPS, Lanthipeptide- Class II	AF	Candicidin	95%
T1PKS	IO, AC	Nigericin	50%
T1PKS	UK	-	ND
T1PKS	UK	-	ND
T1PKS	UK	-	ND
T1PKS	UK	-	ND
T1PKS	UK	-	ND
T1PKS	UK	-	ND
T1PKS	AF	Mediomycin A	28%
T1PKS	UK	-	ND
T2PKS	AF, ATM, AB	Fredericamycin A	60%
T3PKS	ATM	Herboxidiene	11%
NRPS, NRPS-like, terpene	IO, AB	Valinomycin / Montanastatin	13%
NRPS	Q, AB	Streptobactin	76%
NRPS	AB	Dechlorocuracomycin	16%
NRPS	AB	WS9326	7%
NRPS, RRE-containing	AF	Surugamide A / Surugamide D	100%
NRPS	UK	-	ND
Siderophore	AF, AB	Ficellomycin	5%
Siderophore	Q,AF	Desferrioxamin B	100%
Lanthipeptide- Class-III	Mp	AmfS	80%
LAP, Thiopeptide	AF	Fluostatins M-Q	4%
Terpene	St	Hopene	76%
Terpene	AB	Julichrome Q3-3 / Julichrome Q3-5	25%
Terpene	Vo	Geosmin	100%

Terpene, RiPP-like	Pi	Isorenieratene	75%
RiPP-like	UK	-	ND
RiPP-like	UK	-	ND
Butyrolactone, PKS-like, amglyccycl	S	7-deoxypactamycin	15%
Ectoine	Os	Ectoine	100%

*AF, antifungal; AB, antibacterial; INMS, immunosuppressant; PSC, piscicide; UK, unknown; ATM, antitumoral; IO, ionophore; Q, chelator; St, membrane component; Vo, volatile; Pi, pigment; S, signaling; Os, osmolyte; AC, anticarcinogenic; Mp, morphogenic; AM, metabolic activator; T1PKS, polyketide synthase type I; T2PKS, polyketide synthase type II; T3PKS, polyketide synthase type III; NRPS, non-ribosomal peptide synthetase; NRPS-like, NRPS-like fragment; LAP, Linear azol(in)e-containing peptides; NI, NRPS-independent; RRE-containing, RRE-element containing cluster; RiPP-like, other unspecified ribosomally synthesized and post-translationally modified peptide product (RiPP); PKS-like, other types of PKS.

Table S9. Putative BGCs of secondary metabolite from *S. albidoflavus* J29 ori2

**Type**	**Activity**	**Most similar known cluster**	**Similitude percentage (%)**
T1PKS, NRPS-like,NRPS, lanthipeptide Class II	**AF**	Candicidin	100%
T1PKS, NRPS	AF, AB	SGR PTMs/SGR PTM Compound b-d	100%
T1PKS	UK	-	ND
T2PKS, NRPS	AF, ATM, AB	Fredericamycin A	96%
T3PKS	IO, AB	Valinomycin / Montanastatin	34%
NRPS	AM	Dudomycin A	17%
NRPS	AB, AF	Cyclofaulknamycin	75%

NRPS, LAP	AF	Surugamide A / Surugamide D	100%
NRP-metallophore, NRPS	Q	Griseobactin	100%
NI-siderophore	AC	Kinamycin	13%
NI-siderophore	Q	Desferrioxamin B	100%
Lanthipeptide- Class III	AB	SAL-2242	100%
LAP, thiopeptide, RRE-containing	AF	Fluostatins M-Q	4%
Terpene	St	Hopene	76%
Terpene	Vo	Geosmin	100%
Terpene	AB	Julichrome Q3-3 / Julichrome Q3-5	25%
Terpene, NRPS-like, NRPS	IO, AB	Valinomycin / Montanastatin	13%
RiPP-like, terpene	Pi	Isorenieratene	75%
RiPP-like	AF, AB	Hexacosalactone A	4%
RiPP-like	UK	Streptamidine	75%
RiPP-like	UK	-	ND
Butyrolactone, PKS-like, amglyccycl	S	7-deoxypactamycin	15%
Ectoine	Os	Ectoine	100%

* AF, antifungal; AB, antibacterial; INMS, immunosuppressant; PSC, piscicide; UK, unknown; ATM, antitumoral; IO, ionophore; Q, chelator; St, membrane component; Vo, volatile; Pi, pigment; S, signaling; Os, osmolyte; AC, anticarcinogenic; Mp, morphogenic; AM, metabolic activator; T1PKS, polyketide synthase type I; T2PKS, polyketide synthase type II; T3PKS, polyketide synthase type III; NRPS, non-ribosomal peptide synthetase; NRPS-like, NRPS-like fragment; LAP, Linear azol(in)e-containing peptides; NI, NRPS-independent; RRE-containing, RRE-element containing cluster; RiPP-like, other unspecified ribosomally synthesized and post-translationally modified peptide product (RiPP); PKS-like, other types of PKS.
